# The embryonic muscle transcriptome of *Caenorhabditis elegans*

**DOI:** 10.1186/gb-2007-8-9-r188

**Published:** 2007-09-12

**Authors:** Rebecca M Fox, Joseph D Watson, Stephen E Von Stetina, Joan McDermott, Thomas M Brodigan, Tetsunari Fukushige, Michael Krause, David M Miller

**Affiliations:** 1 Department of Cell and Developmental Biology, Vanderbilt University, 465 21^st ^Ave. S., Nashville, TN 37232-8240, USA; 2 Graduate Program in Neuroscience, Center for Molecular Neuroscience, Vanderbilt University, Nashville, TN 37232-8548, USA; 3 Laboratory of Molecular Biology, National Institute of Diabetes, Digestive and Kidney Diseases, National Institutes of Health, Building 5, Room B1-04, Bethesda, MD 20892, USA; 4 Current address: Department of Cell Biology, Johns Hopkins University School of Medicine, 725 N. Wolfe St., Baltimore, MD 21205, USA

## Abstract

**Background:**

The force generating mechanism of muscle is evolutionarily ancient; the fundamental structural and functional components of the sarcomere are common to motile animals throughout phylogeny. Recent evidence suggests that the transcription factors that regulate muscle development are also conserved. Thus, a comprehensive description of muscle gene expression in a simple model organism should define a basic muscle transcriptome that is also found in animals with more complex body plans. To this end, we applied microarray profiling of *Caenorhabtidis elegans *cells (MAPCeL) to muscle cell populations extracted from developing *C. elegans *embryos.

**Results:**

We used fluorescence-activated cell sorting to isolate *myo-3*::green fluorescent protein (GFP) positive muscle cells, and their cultured derivatives, from dissociated early *C. elegans *embryos. Microarray analysis identified 7,070 expressed genes, 1,312 of which are enriched in the *myo-3*::GFP positive cell population relative to the average embryonic cell. The muscle enriched gene set was validated by comparisons with known muscle markers, independently derived expression data, and GFP reporters in transgenic strains. These results confirm the utility of MAPCeL for cell type specific expression profiling and reveal that 60% of these transcripts have human homologs.

**Conclusion:**

This study provides a comprehensive description of gene expression in developing *C. elegans *embryonic muscle cells. The finding that more than half of these muscle enriched transcripts encode proteins with human homologs suggests that mutant analysis of these genes in *C. elegans *could reveal evolutionarily conserved models of muscle gene function, with ready application to human muscle pathologies.

## Abstract 2

Fluorescence activated cell sorting and microarray profiling were used to identify 1,312 expressed genes that are enriched in *myo-3*::GFP-positive muscle cells of *Caenorhabditis elegans*.

## Background

The basic architecture of the muscle contractile unit, the sarcomere, and regulatory processes that control muscle activity are remarkably similar in motile animals. For example, sarcomeres are universally assembled from interdigitating myosin thick filaments and actin thin filaments; this complex is activated by intracellular calcium to drive muscle contraction [1-3]. In addition to these important functional and structural elements, transcription factors that direct muscle differentiation are also conserved. In mammals, a group of basic helix-loop-helix transcription factors or myogenic regulatory factors (MRFs) define a transcriptional cascade that directs skeletal muscle differentiation [4]. A similar pathway functions in the nematode, *Caenorhabtidis elegans*, in which a single MRF-related factor, HLH-1 (helix-loop-helix), is highly expressed in all embryonic body wall muscle cells [5,6]. A determinative role of HLH-1 in embryonic muscle differentiation is suggested by the finding that ectopic HLH-1 is sufficient to convert other embryonic cell types to a body wall muscle fate. Interestingly, body wall muscle differentiation in *C. elegans *also depends on two other transcription factors, namely UNC-120 (serum response factor) and HND-1 (HAND family of basic helix-loop-helix factors), conserved homologs of which are selectively required for vertebrate smooth muscle and cardiac muscle differentiation, respectively. This finding suggests that vertebrate muscles may have arisen from a common primordial invertebrate muscle cell [7]. It follows that pathways that define *C. elegans *body wall muscle differentiation and function may be encoded by genes that contribute to all three major classes of vertebrate muscles.

In *C. elegans*, 81 body wall muscle cells are generated before hatching to comprise the predominant embryonic muscle cell type. Minor embryonic muscles include two anal muscles and two myoepithelial cells that envelope the posterior intestine [1,3]. All of these muscles express the myosin heavy chain gene *myo-3 *(myosin heavy chain 3) [8]. A distinct group of 20 muscle cells in the feeding organ or pharynx are also generated in the embryo but they do not express *myo-3*.

**Figure 1:**
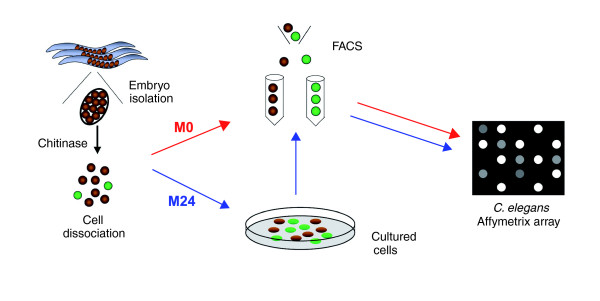
Profiling strategy for *myo-3*::GFP muscle cells. Embryos are released from gravid adults and dissociated with chitinase. *myo-3*::green fluorescent protein (GFP) labeled muscle cells (green) were isolated by fluorescence-activated cell sorting (FACS) directly from freshly dissociated embryos to generate a profile of nascent body muscle cells (M0) and from embryonic cells after 24 hours in culture to obtain microarray data from fully differentiated muscle cells (M24). RNA extracted from each set of isolated muscle cells was amplified and labeled for hybridization to *C. elegans *whole genome Affymetrix arrays. GFP, green fluorescent protein.

Extensive genetic screens have identified large numbers of mutations that disrupt the structure and organization of body wall muscle cells [9-12]. Although this approach has revealed key molecules (for instance, *myo-3*) with important roles in muscle function and development, the complexity of these processes suggests that many additional *C. elegans *genes are also likely to contribute to the myogenic program [13]. Here we describe the application of a recently developed technique, microarray profiling of *C. elegans *cells (MAPCeL), to generate a comprehensive catalog of *C. elegans *genes expressed in embryonic body wall muscle cells. In this method, cells marked with a specific green fluorescent protein (GFP) reporter gene are isolated by fluorescence-activated cell sorting (FACS) for microarray profiling experiments [14]. The sorted cells can be obtained either from freshly dissociated embryos, in which early developmental genes are expressed, or from mature cells after differentiation in culture (Figure 1). Thus, this approach can potentially identify distinct sets of genes that may respond to extrinsic signals that influence cell fate and differentiation in the early embryo as well as transcripts that are expressed later in development as the sarcomere apparatus begins to function. We have used the *myo-3*::GFP reporter gene to mark nonpharyngeal embryonic muscle cells in *C. elegans *[15]. This robust reporter initiates expression during early embryonic myogenesis and also perdures in mature embryonic muscle cells. We have exploited the continuous embryonic expression of *myo-3*::GFP to profile *C. elegans *body wall muscle cells at these two developmental stages. In addition to revealing genes that are differentially expressed in these distinct myogenic populations, this approach has also identified transcripts, such as *myo-3*, that are enriched in muscle cells throughout embryonic development. A common group of about 600 genes in these datasets are also upregulated in an independent microarray profile of HLH-1 induced transcripts in the *C. elegans *embryonic cells [7]. This overlapping set of MRF-regulated mRNAs defines a core group of candidate genes with potentially key roles in muscle development and function. In the future, analysis of these gene sets with the facile genetic tools available in this model organism should lead to a detailed understanding of the logic of the muscle transcriptome and its role in myofilament assembly and function.

## Results

### Strategy to profile *C. elegans *embryonic body wall muscle cells

**Figure 2:**
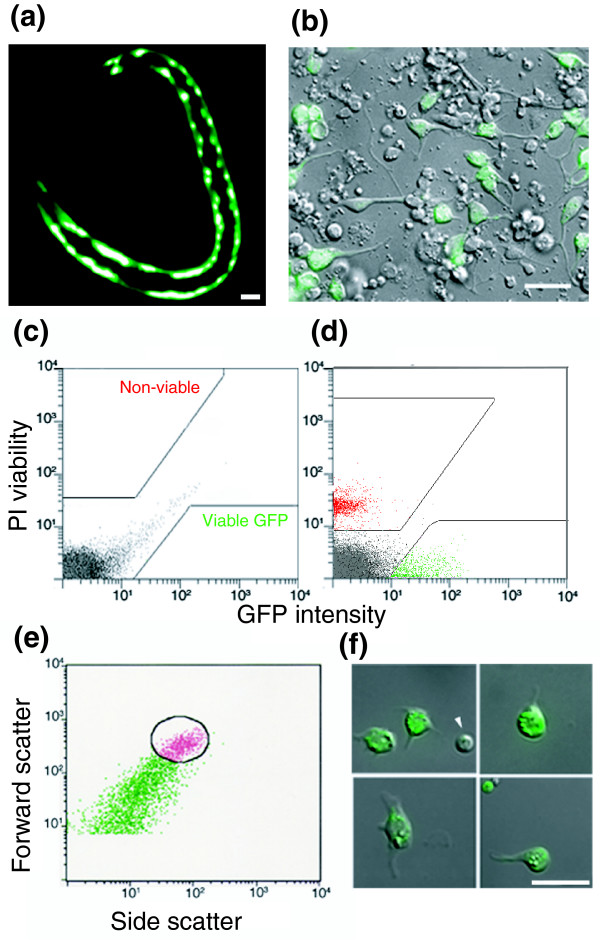
Isolation of *myo-3*::GFP muscle cells by FACS. **(a) ***myo-3*::green fluorescent protein (GFP) expression in the body wall muscle cells of a newly hatched L1 larva. **(b) **Combined DIC and fluorescence image of a 24-hour culture of *myo-3*::GFP muscle cells. Panels c to e show fluorescence-activated cell sorting (FACS) profiles. **(c) **Fluorescence intensity scatter plot of wild-type (non-GFP) cells. Boxed areas exclude autofluorescent cells (gray). **(d) ***myo-3*::GFP cells (green) are gated to exclude propidium iodide (PI) stained cells (red). **(e) **Light scattering gate for GFP-positive cells (circle) to exclude cell clumps and debris. **(f) ***myo-3*::GFP muscle cells after enrichment by FACS. Scale bars: 5 μm.

We used MAPCeL [14] to assess mRNA expression in embryonic muscle cells. This technique involves the dissociation of blastomeres from embryos expressing a cell-type restricted GFP reporter gene, thus allowing FACS-enrichment of specific cell types (Figure 1). To mark embryonic muscle cells, we used an integrated *myo-3*::GFP transgene [15]. *myo-3*::GFP expression begins early, in the 'pre-comma' stage embryo that is readily dissociated into individual blastomeres. This reporter is expressed in all 81 embryonic body wall muscle cells (Figure 2), the anal depressor, and sphincter muscles. We used MAPCeL to profile muscle cells from two cell populations (Figure 2) [16]: *myo-3*::GFP labeled blastomeres sorted directly from freshly dissociated embryos; and *myo-3*::GFP expressing muscle cells from dissociated embryos cultured for 24 hours before sorting.

The microarray profile of freshly dissociated muscle cells is labeled 'M0' to denote direct isolation from embryos at '0' hours (normalized intensity values are listed in Additional data file 1). The M0 profile is expected to include transcripts that are highly expressed in nascent muscle cells. The embryonic *myo-3*::GFP positive body wall muscle cells comprise about 15% of the total cell population (81/550 total cells), which is consistent with the frequency at which *myo-3*::GFP expression is detected in dissociated embryonic cells (Figure 2b) [17].

*myo-3*::GFP expression persists in fully differentiated muscle cells after the comma stage, when embryos become resistant to dissociation. We have previously shown that *C. elegans *neurons and muscle cells can differentiate *in vitro *from early embryonic blastomeres [16,17]. Therefore, to obtain a profile of mature embryonic muscle cells, dissociated *myo-3*::GFP embryos were cultured for 24 hours before sorting; the microarray dataset from these *myo-3*::GFP cells is labeled 'M24' (Additional data file 1). mRNAs in the M24 profile are expected to represent transcripts expressed in differentiated body wall muscle cells. Although *myo-3*::GFP is also expressed in post-embryonically derived muscle cells (for instance, vulval), larval cells apparently do not differentiate under these culture conditions and therefore should not be directly profiled in these experiments [17].

### Microarray profiles are reproducible

**Figure 3:**
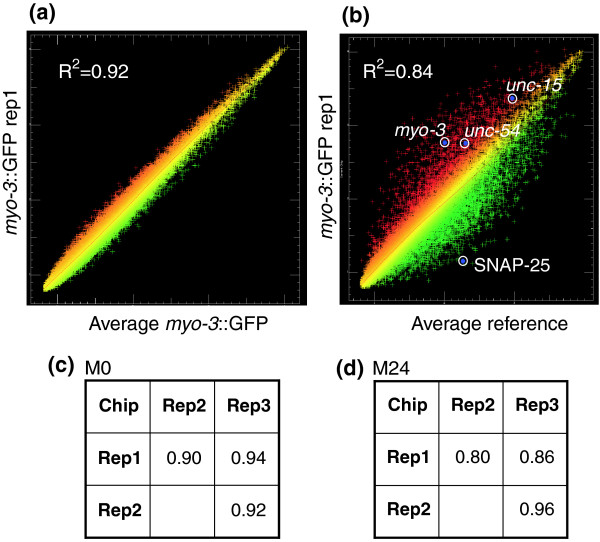
Coefficients of determination (*R*^2^) for individual hybridizations. **(a) **Scatter plot of a representative hybridization of a single *myo-3*::green fluorescent protein (GFP) replicate (Rep1) to the average intensities for all three *myo-3*::GFP (M0) hybridizations. **(b) **Results of a single *myo-3*::GFP hybridization (red) compared with average reference intensities (green) to identify transcripts exhibiting differential expression. Known muscle genes *unc-54*, *myo-3*, and *unc-15 *(top circles) are enriched in *myo-3*::GFP muscle cells, whereas the neuronal transcript encoding SNAP-25 is depleted (bottom circle). **(c) ***R*^2 ^values for pair-wise comparisons of *myo-3*::GFP M0 datasets (average = 0.92). **(d) ***R*^2 ^values for pairwise *myo-3*::GFP M24 datasets (average = 0.87).

The coefficient of determination (*R*^2^) was calculated for each set of microarray replicates. An average *R*^2 ^of 0.94 (*n *= 3) was obtained for the reference dataset (R0) obtained from freshly dissociated embryonic cells. The reproducibility of these data is illustrated graphically in the representative scatter plot shown in Figure 3. A similar high value of *R*^2 ^(0.96; *n *= 4) was previously determined for the reference data (R24) obtained from all embryonic cells after 24 hr in culture [14]. *R*^2 ^values for pair-wise combinations of the M0 (average *R*^2 ^= 0.92) and M24 (average *R*^2 ^= 0.87) datasets are shown in Figure 3.

### Detecting expressed genes in muscle cells

**Figure 4:**
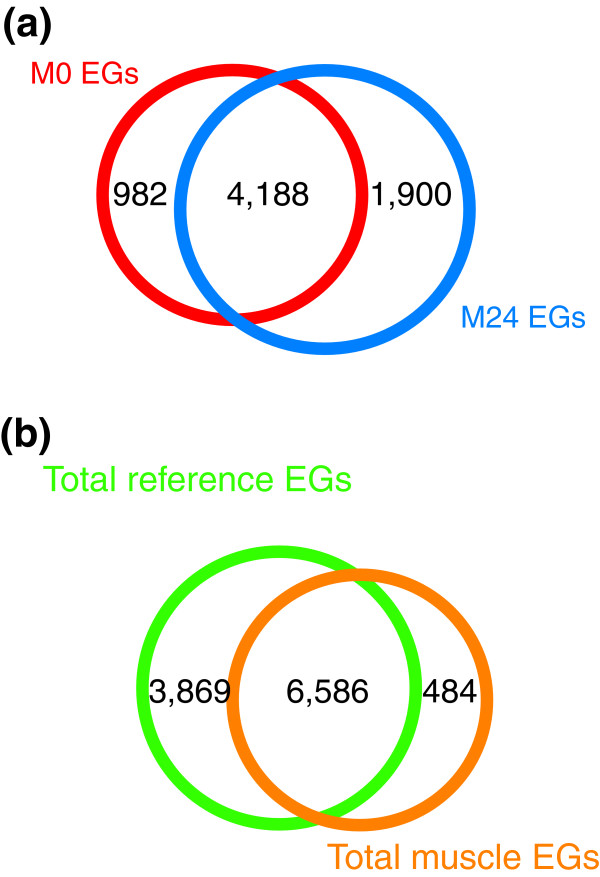
Comparison of expressed genes in muscle and reference datasets. **(a) **A total of 7,070 expressed genes (EGs) are detected in the M0 and M24 profiles of body wall muscle cells, of which 4,188 are common to both datasets. The M0 profile contains 982 genes that are not expressed in the M24 dataset, whereas 1,900 transcripts are exclusively detected in the M24 profile. **(b) **The combined muscle and reference datasets include 10,939 EGs. Of these transcripts, 6,586 are detected in all datasets whereas 484 genes are exclusive to the combined muscle datasets and 3,869 selectively detected in the reference profiles of all embryonic cells.

We initially identified all transcripts that are reliably detected in the muscle datasets. These lists of 'present' genes for the experimental M0 and M24 datasets were adjusted to remove transcripts that could easily be attributed to contamination by non-GFP cells (about 10%) in FACS-derived *myo-3*::GFP cell populations (see Materials and methods, below) [14]. The resultant list of 'expressed genes' includes 7,070 unique mRNAs from the M0 and M24 populations of *C. elegans *body wall muscle cells (Figure 4a and Additional data file 2). A total of 10,455 unique expressed genes are included in the sum (R0 + R24) of the reference datasets; overall, 10,939 transcripts were detected in these experiments. A substantial number of expressed genes (6,586) are expressed in both muscle cells and in the reference dataset (Figure 4b and Additional data file 3). These transcripts are likely to include 'housekeeping' genes that play universal roles in cell differentiation and homeostasis; for example, transcripts for 75 ribosomal proteins are included in this group (Additional data file 3). Expressed genes that are selectively detected in the M0 and M24 profiles are likely to provide functions that are largely restricted to muscle cells (Figure 4b and Additional data file 3). These 'muscle-specific' genes, as well as transcripts showing 'enriched' expression in muscle cells relative to other embryonic cells, are described in detail below.

### Microarray profiles detect muscle-enriched transcripts

A scatter plot comparing the M0 muscle dataset with the R0 reference reveals significant differences in gene expression levels (Figure 3b). Enrichment for known muscle genes is evident, because transcripts for the abundant muscle structural proteins MYO-3 (myosin heavy chain), UNC-54 (myosin heavy chain), and UNC-15 (paramyosin) [18-20] are highly elevated (red) relative to reference data obtained from all embryonic cells. Other transcripts, including those encoding SNAP-25 (a synaptic vesicle protein expressed in neurons) [21], are depleted (green; Figure 3b). A similar scatter plot was obtained for a comparison of the M24 muscle and R24 reference profiles (data not shown).

**Figure 5:**
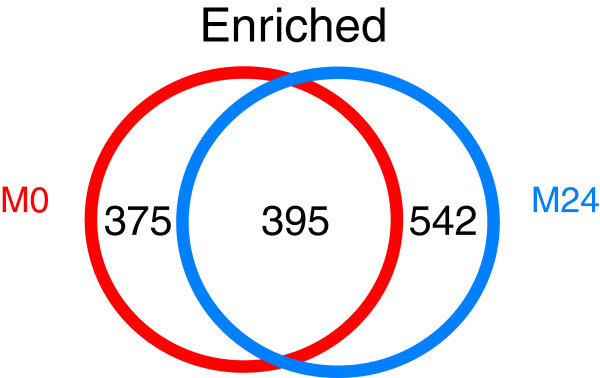
Comparison of enriched transcripts in the M0 and M24 *myo-3*::GFP datasets. A total of 395 transcripts are enriched in both datasets; 375 genes are exclusive to the M0 dataset and 542 are selectively enriched in M24. A total of 1,312 transcripts are enriched in body wall muscle cells compared to reference cells.

Transcripts that are differentially expressed in the M0 and M24 muscle datasets were identified by a statistical comparison of the paired experimental and reference datasets (for instance, M0 versus R0 and M24 versus R24; see Materials and methods, below). This treatment identified a total of 770 genes that are significantly enriched in the M0 muscle dataset and 937 transcripts with elevated expression relative to reference in the M24 profile. A comparison of these data identified 1,312 unique transcripts that are enriched in at least one of these datasets (Figure 5 and Additional data file 4). Conversely, 2,542 genes are depleted in embryonic body wall muscles in comparison with all cells (Additional data file 5).

### Validation of transcripts detected in body muscle profiles

A survey of the literature and a comprehensive search of WormBase [22] (see Materials and methods, below) identified 1,003 genes with known expression in *myo-3*::GFP-positive embryonic muscle cells (body wall muscle and defecation muscles; Additional data files 6 and 7; also see Materials and methods, below). A majority of these genes (773/1,003 [77%]) are detected as expressed genes in *myo-3*::GFP muscle cells (Additional data file 2). In contrast, only 28% (1,003/3,544) of all genes with expression patterns listed in WormBase are annotated as expressed in muscle (Additional data file 5).

Consistent with the low false discovery rate calculated for these datasets, we detected limited overlap with microarray profiles generated from other cell types. For example, only 100 out of 1,685 intestine or germline-enriched transcripts [23] are also listed in our enriched muscle dataset (Additional data file 8). These intestine and germline genes are thus under-represented in the embryonic muscle profile (representation factor = 0.8, *P *< 0.036). (Hypergeometric calculations were performed as described by Von Stetina and coworkers [24].) In contrast, a similar comparison of the embryonic muscle enriched genes detected significant overlap with transcripts that are also elevated in a MAPCeL dataset obtained from embryonic A-class motor neurons [14]. In this case 159 of the approximately 1,000 embryonic A class motor neuron enriched transcripts are detected in the muscle profile (representation factor = 2.3, *P *< 1.5 × e^-24^; Additional data file 9). The significantly higher fraction of shared transcripts between neurons and muscles could be indicative of the common functions of excitable cells. For example, transcripts for the acetylcholine receptors (*unc-38 *and *unc-63*), ryanodine calcium receptor (*unc-68*), and innexin gap junction protein (*unc-9*) are detected in both the muscle and A-class motor neuron datasets. This view is consistent with the finding that the embryonic muscle dataset also shows significant overlap with a MAPCeL profile of the *C. elegans *embryonic nervous system (representation factor = 2.0, *P *< 2.5 × e^-24^; Additional data file 7) [24].

**Figure 6:**
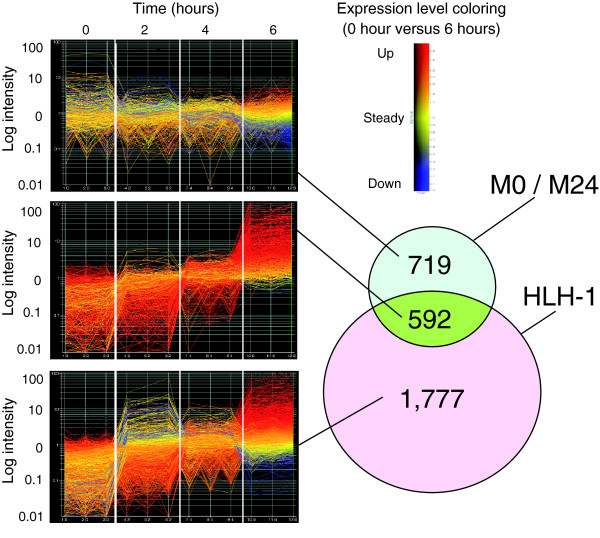
Comparison of M0 and M24 enriched transcripts to HLH-1 induced muscle genes. Embryos in which most blastomeres have been converted to muscle-like cells by the induced expression of an *hlh-1 *transgene were profiled over time for gene expression [7]. Data were obtained from the Affymetrix platform also used for the M0 and M24 profiles, allowing a direct comparison of the datasets. The Venn diagram shows the overlap between the M0 + M24 and the HLH-1 induced transcripts with at least a 1.7-fold increase in expression compared with the respective reference samples. Panels to the left show the time course of gene expression (GeneSpring software; Agilent) for three independent samples at each time point for the HLH-1 induced dataset. Line coloring in these graphs reflects the 6-hour value compared with the 0 hour value for each gene, as indicated by the color key. The 592 transcripts common to both experimental approaches are strong candidates for muscle specific genes; most of these show induction (up to 100-fold) in the HLH-1 induced dataset.

Two previous studies, using different methodologies, have also reported body wall muscle gene expression, and these can serve as validation tests for our methods. Fukushige and coworkers [7] used the same microarray platform (Affymetrix) to examine body wall muscle-like gene expression resulting from nearly uniform myogenic conversion of early *C. elegans *blastomeres by the transcription factor HLH-1 (CeMyoD). Of the 1,312 transcripts that are enriched in at least one of the embryonic MAPCeL muscle datasets, 592 (about 45%) are upregulated in body wall muscle-like cells at 6 hours post-induction of HLH-1 (representation factor = 3.6, *P *< 6.5 × e^-205^; Figure 6 and Additional data file 8). This finding is clearly indicative of highly similar muscle profiles. In contrast, the MAPCeL list of embryonic muscle enriched genes shows less overlap with a microarray profile of larval body muscle cells obtained by the mRNA-tagging method [25], although the 249 transcripts shared by both datasets are indicative of significant similarity (representation factor = 2.8, *P *< 1.8 × e^-54^; Additional data file 6). It is unclear whether this disparity is due to the different profiling strategies used to generate these data or to developmentally regulated differences in gene expression between embryonic and larval muscles.

The finding that a majority of known muscle genes is detected in our microarray profiles, and that these datasets exhibit substantial overlap with an independent profile of embryonic myogenesis [7] suggested that other uncharacterized transcripts in these datasets are also likely to be expressed in body wall muscle cells. To test this idea, we generated promoter-GFP reporter genes for representative transcripts in the M0 and M24 datasets and scored expression in embryonic and post-embryonic muscle cell types. A 'promoter' was defined as the region upstream of the ATG start codon for a distance of 4 kilobases or the distance to the end/beginning of the 5' flanking gene, whichever was less. In some cases, the promoter region tested was quite small (as little as 450 base pairs) and therefore may not have included necessary regulatory elements for expression of the transgene (Additional data file 11).

We found that about 70% (36/52) of transgenic lines generated from these reporter genes exhibited GFP-positive muscle cells *in vivo *(Additional data file 11). This finding is comparable to the finding that 61% (238/393) of genes in the total muscle enriched dataset for which expression patterns are listed in WormBase are annotated as expressed in muscle. In contrast, only 28% (1,003/3,544) of all genes with expression patterns in WormBase are identified as muscle expressed (Additional data files 6 and 7). The majority of muscle positive promoters (20/36) drove expression in both embryonic and post-embryonic muscle, although 16 had no detectable embryonic expression. We saw no correlation between the rank order of transcripts identified by MAPCeL and the likelihood of muscle expression of the corresponding GFP reporters, suggesting that these microarray datasets are robust (Additional data file 11).

**Figure 7:**
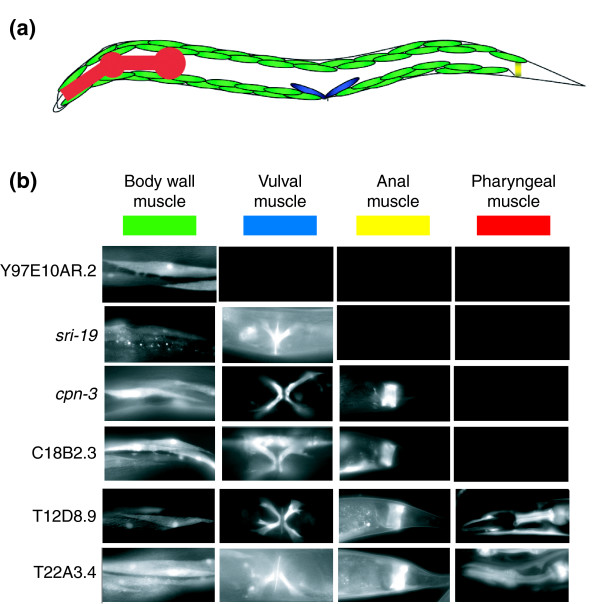
GFP reporters verify muscle genes. **(a) **Schematic showing major muscle groups of *C. elegans*. *myo-3*::green fluorescent protein (GFP) is expressed in body wall muscle (green), vulval muscle (blue), and anal muscle (yellow). Pharyngeal muscle is shown in red. **(b) **Expression of representative GFP reporters. Gene names are shown on the left.

**Figure 8:**
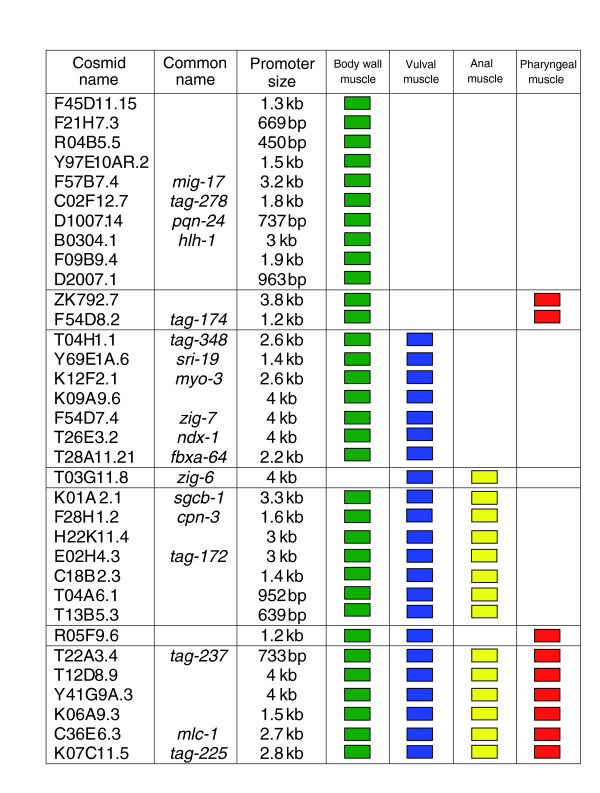
Comprehensive list of GFP reporters generated in this study showing expression in muscle cells.

Figure 7a depicts expression of representative GFP reporters in three *myo-3*::*GFP *positive muscle cell types (body wall, vulval, and defecation) and pharyngeal muscle as scored in late larvae and adults. Given that body wall cells are the predominant muscle cell type, it is not surprising that most (35/36) of the muscle positive reporters showed expression in this tissue. The one exception, *zig-6*::GFP, is detected in embryonic anal muscles, a finding that underscores the sensitivity of our methods to transcripts that may be selectively expressed in a subset of embryonically generated muscles. Twenty-two GFP reporters were also expressed in the vulval muscles, although these post-embryonically derived cells are likely to be absent from primary cultures [17] and therefore were not directly profiled by our methods (Figures 7b and 8). This finding must reflect underlying similarities between vulval and body wall muscle cells. Interestingly, six reporters show expression in all four muscle types and may be indicative of genes required for general muscle function (Figure 8). It is noteworthy that a majority of the corresponding endogenous genes for the body wall muscle positive GFP reporters (31/36 [86%]) were also strongly upregulated (≥1.7 fold) during HLH-1 induced embryonic myogenesis (Figure 6). In comparison, only 19% of muscle negative GFP reporters (3/16) exhibit similar upregulation (Additional data file 11). The analysis of GFP reporters constructed from the muscle enriched datasets confirms muscle expression *in vivo *and also potentially reveals interesting examples of genes with roles common to all four major muscle types as well as other transcripts with functions that may be selectively required in specific subsets of body muscle cells.

### Detection of transcripts that are differentially expressed in nascent (M0) versus differentiated (M24) body wall muscle cells

The experiments performed in this study profile muscle cells that presumptively differ in developmental age. The M0 dataset is comprised of early pre-morphogenesis embryonic cells whereas the M24 dataset includes muscle cells that have differentiated in culture for 24 hours. A comparison of transcripts enriched in both datasets reveals 401 common genes (Figure 5). Interestingly, of 38 transcripts encoding muscle structural proteins, 74% (28/38) are common to both datasets (Additional data file 12). This finding indicates that other genes in this list of 395 transcripts may also fulfill key roles in both nascent and fully differentiated muscle cells, and may therefore constitute a class of fundamental muscle function genes.

In addition to transcripts that are elevated in both datasets, we also detected genes that are selectively enriched in either the M0 or M24 profiles. Overall, 375 genes show elevated expression in the M0 dataset only whereas a separate group of 542 transcripts are exclusively enriched relative to all other cells in the M24 dataset (Figure 5). Of genes that are differentially detected in these datasets, we note that *pat-3 *and *pat-6*, which are required for initial muscle assembly [11,26,27], are selectively enriched in the M0 profile. Conversely, *unc-70 *is detected as an expressed gene in the M0 dataset but it is exclusively elevated in the M24 profile, a result that is consistent with the finding that UNC-70 (β-spectrin) is expressed in all embryonic cells early in development but is localized to muscles and neurons at hatching [28]. It is also possible that some of these differences could be induced by differences in the cellular environments of the M0 (intact embryo) and M24 (*in vitro *culture) muscle cells. For example, 24 genes encoding proteosome subunits show elevated expression in the M24 dataset whereas none of these transcripts are enriched in the M0 profile. This finding could be indicative of the general lack of innervation of muscle cells in culture because the removal of motor neuron activity *in vivo *results in increased muscle protein degradation via a proteosome dependent mechanism [29]. Despite this caveat, these MAPCeL data appear to reveal differences in gene expression that correlate with the developmental 'age' of the M0 and M24 muscle cell populations, suggesting that this technique may be generally useful for detecting temporal changes in gene expression during development.

### Gene families enriched in muscle cells

Genetic studies in *C. elegans *have identified a large number of genes that are required for muscle structure, development, and function [2,3]. To assess the potential utility of our microarray data for expanding this catalog of muscle genes, we organized transcripts in these profiles according to functional categories. A sampling of these findings is presented below. Genes exhibiting enriched transcript levels are highlighted in bold when they are first identified in the text. All genes discussed in this section are listed in Table 1.

**Table 1:** Gene families enriched in muscle cells

	Cosmid Name	Common Name	Rank M0	Rank M24	KOG (or other description)
Muscle	K12F2.1	*myo-3*	710	240	Myosin class II heavy chain
structure and function	F11C3.3	*unc-54*	-	431	Myosin class II heavy chain
	Y11D7A.14		-	826	Myosin class II heavy chain
	F45G2.2		-	743	Myosin class II heavy chain
	C36E6.3	*mlc-1*	93	156	Myosin regulatory light chain, EF-Hand protein superfamily
	C36E6.5	*mlc-2*	329	189	Myosin regulatory light chain, EF-Hand protein superfamily
	F09F7.2	*mlc-3*	279	246	Myosin essential light chain, EF-Hand protein superfamily
	F07A5.7	*unc-15*	18	298	Myosin class II heavy chain
	ZK617.1	*unc-22*	493	245	Projectin/twitchin and related proteins
	W06H8.8	*ttn-1*	168	534	Projectin/twitchin and related proteins
	F54E2.3	*ketn-1*	6	154	Unnamed Protein (Invertebrate Titin-like protein)
	K03E6.6	*pfn-3*	64	94	Profilin
	K06A4.3		584	389	Actin regulatory proteins (gelsolin/villin family)
	Y71G12B.11		4	122	Talin
	W03F11.6	*afd-1*	-	393	Actin filament-binding protein Afadin
	F08A8.6	*tag-138*	-	474	Actin-binding protein SLA2/Huntingtin-interacting protein Hip1
	Y66H1B.3		462	784	Actin-binding cytoskeleton protein, filamin
	Y66H1B.2		119	902	Actin-binding cytoskeleton protein, filamin
	R01H10.3	*cor-1*	766	20	Actin-binding protein Coronin, contains WD40 repeats
	Y105E8B.1	*lev-11*	530	508	Actin filament-coating protein tropomyosin
	F42E11.4	*tni-1*	120	215	Troponin I
	ZK721.2	*unc-27*	236	111	Troponin I
	T20B3.2	*tni-3*	311	235	Troponin I
	T22E5.5	*mup-2*	230	166	Troponin
	C14F5.3	*tnt-3*	173	281	Troponin
	ZC477.9	*deb-1*	13	271	Alpha-catenin
	ZK1058.2	*pat-3*	99	-	Integrin beta subunit (N-terminal portion of extracellular region)
	C29F9.7	*pat-4*	428	401	Integrin-linked kinase
	F54C1.7	*pat-10*	184	53	Calmodulin and related proteins (EF-Hand superfamily)
	C47E8.7	*unc-112*	436	559	Mitogen inducible gene product (contains ERM and PH domains)
	C18A11.7	*dim-1*	108	74	Immunoglobin and related proteins
	K11C4.5	*unc-68*	438	318	(Ryanodine receptor, Ca^2+ ^release channel)
	K11D9.2	*sca-1*	192	31	Ca^2+ ^transporting ATPase
	C18E9.1	*cal-2*	470	547	Calmodulin and related proteins (EF-Hand superfamily)
	C54E10.2		-	578	Ca^2+ ^sensor (EF-Hand superfamily)
	F21A10.1		760	650	Ca^2+ ^sensor (EF-Hand superfamily)
	K03E6.3	*ncs-3*	659	185	Ca^2+ ^sensor (EF-Hand superfamily)
	F40E10.3	*csq-1*	157	234	Calsequestrin
Dystrophin glycoprotein complex	F15D3.1	*dys-1*	265	869	Dystrophin-like protein
	C33G3.1	*dyc-1*	187	774	Muscular protein implicated in muscular dystrophy phenotype
	F30A10.8	*stn-1*	626	476	Syntrophins (type beta)
	F07H5.2	*sgn-1*	275	121	Gamma/delta sarcoglycan
	K01A2.1	*sgcb-1*	210	640	(Beta-sarcoglycan)
	H22K11.4		415	35	Sarcoglycan complex, alpha/epsilon subunits
	F27D9.8	*stn-2*	92	-	Syntrophin (type gamma)
	T03F7.1	*snf-11*	507	644	(Sodium-neurotransmitter symporter)
Transcription factors	Y47D3B.7	*sbp-1*	326	7	Predicted DNA-binding protein
	F46G10.6	*mxl-3*	556	8	Upstream transcription factor 2/L-myc-2 protein
	T20B3.3	*srh-215*	-	52	Predicted olfactory G-protein coupled receptor
	T26C11.1	*tbx-41*	-	127	TBX2 and related T-box transcription factors
	T04C10.4	*atf-5*	-	175	Activating transcription factor 4
	C29G2.5	*srt-58*	-	314	7-transmembrane receptor
	F40G9.11	*mxl-2*	-	315	bHLHZip transcription factor BIGMAX
	W07G1.3	*zip-3*	-	352	Activating transcription factor 4
	D1081.2	*unc-120*	353	331	Regulator of arginine metabolism and related MADS box-containing transcription factors
	T27C4.4	*egr-1*	-	346	Histone deacetylase complex, MTA1 component
	Y46H3D.6	*nhr-237*	-	432	Hormone receptors
	C33G8.6	*nhr-42*	-	515	Hormone receptors
	C49D10.2	*nhr-166*	-	529	Nuclear hormone receptor
	T24A6.11	*nhr-222*	-	527	Nuclear hormone receptor
	F45E4.9	*hmg-5*	-	540	HMG box-containing protein
	H05G16.1	*frm-3*	-	655	Rho guanine nucleotide exchange factor CDEP
	T22H6.6	*gei-3*	-	643	HMG-box transcription factor Capicua and related proteins
	C10G8.7	*ceh-33*	608	670	Transcription factor SIX and related HOX domain proteins
	C10G8.6	*ceh-34*	354	671	Transcription factor SIX and related HOX domain proteins
	B0414.2	*rnt-1*	91	667	Runt and related transcription factors
	Y46H3D.7	*nhr-238*	-	695	Hormone receptors
	ZC64.3	*ceh-18*	-	685	Transcription factor OCT-1, contains POU and HOX domains
	F44E2.6		-	746	Predicted pilin-like transcription factor
	F41B5.9	*nhr-182*	-	849	Hormone receptors
	B0304.1	*hlh-1*	142	83	Myogenic helix-loop-helix transcription factor
	R13A5.5	*ceh-13*	324	-	Transcription factor zerknullt and related HOX domain proteins
	F28B12.2	*egl-44*	451	-	TEF-1 and related transcription factor, TEAD family
	T14G12.4	*fkh-2*	433	-	Transcription factor of the Forkhead/HNF3 family
	F38A6.3	*hif-1*	564	-	Hypoxia-inducible factor 1/Neuronal PAS domain protein NPAS1
	C37F5.1	*lin-1*	25	-	Predicted transcription factor
	R03E9.1	*mdl-1*	378	-	Upstream transcription factor 2/L-myc-2 protein
	Y5H2B.2	*nhr-13*	594	-	Hormone receptors
	F58G6.5	*nhr-34*	715	-	Hormone receptors
	T23H4.2	*nhr-69*	186	-	Hepatocyte nuclear factor 4 and similar steroid hormone receptors
	Y68A4A.6	*srz-96*	625	-	7-transmembrane receptor
	K10D3.3	*taf-11.2*	452	-	Transcription initiation factor TFIID, subunit TAF11
	Y65B4BR.5		250	-	Transcription factor containing NAC and TS-N domains
	K06A9.2		518	-	(F-box protein, contains homeobox domain)
Neuromuscular junction	F09E8.7	*lev-1*	631	173	Acetylcholine receptor
	C35C5.5	*lev-8*	84	757	Acetylcholine receptor
	T08G11.5	*unc-29*	70	400	Acetylcholine receptor
	F21F3.5	*unc-38*	441	427	Acetylcholine receptor
	Y110A7A.3	*unc-63*	-	78	Acetylcholine receptor
	ZC504.2	*acr-8*	623	749	Acetylcholine receptor
	F25G6.3	*acr-16*	-	42	Acetylcholine receptor
	T21C12.1	*unc-49*	416	-	GABA receptor
	K06C4.6	*mod-1*	-	806	Ligand-gated ion channel
	C27H5.8	*glc-4*	770	-	Ligand-gated ion channel
	T24D8.1		-	65	Ligand-gated ion channel
	T27A1.4		199	807	Ligand-gated ion channel
	Y113G7A.5		752	-	Ligand-gated ion channel
	F22A3.3	*glr-8*	248	-	Glutamate-gated kainate-type ion channel receptor subunit GluR5 and related subunits
	ZK867.2		593	-	(similar to glutamate receptor)

### Muscle structure and function

**Figure 9:**
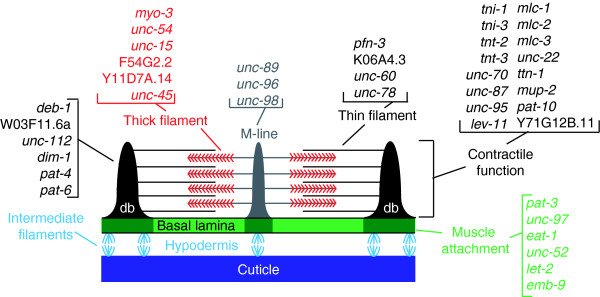
Key sarcomere components are enriched in microarray profiles of embryonic muscle cells. Schematic diagram of a sarcomere showing thin filament links to dense bodies (db) and thick filament attachments to the M-line. Molecular identities of indicated genes (for example, *deb-1*, W03F11.6a, and so on) are presented in the Table 1, Additional data file 3, and in the text. Adapted from Moerman and Williams [2].

The overall organization of *C. elegans *body wall muscle cells is similar to that of vertebrate skeletal muscle. The primary functional component is the sarcomere, a structure composed of myosin-containing thick filaments (A-band) that interdigitate with actin-containing thin filaments (I-band). The nematode sarcomere resembles vertebrate striated muscle, although it is obliquely striated with myosin and actin-containing filaments oriented at an angle of 6° with respect to sarcomere end plates [1,2,30]. The sarcomere maintains functional alignment through attachment of thin filaments to dense bodies, which link thin filaments to the basement membrane of the cell. Thick filaments are stabilized within the sarcomere by the M-line, a specialized region in the A-band that links adjacent thick filaments. The dense bodies and the M-line are the primary mediators of tension generated during muscle contraction [10]. Hemidesmosomes that connect each muscle cell to the overlying cuticle transmit this force to deform the exoskeleton and thereby propel locomotion [30,31] (Figure 9).

Thick filaments are largely comprised of two myosin heavy chain (MHC) proteins, MHC A and MHC B, encoded by the ***myo-3 ***and ***unc-54 ***genes, respectively [1,8,20]. Interestingly, *myo-3 *is enriched in both the M0 and M24 datasets whereas *unc-54 *is selectively elevated in the M24 profile but detected as an expressed gene in M0 muscle cells. The elevation of *myo-3 *transcript levels before *unc-54 *mRNA during body wall muscle development is consistent with the observation that MHC A protein is also more abundant than UNC-54 in early embryonic muscle cells [32]. The apparent sequential expression of *myo-3 *and *unc-54 *parallels their distinct roles in thick filament assembly; MHC A establishes a bipolar nucleation complex to which UNC-54 is added as the filament elongates [8,33,34]. Differential roles in muscle development are also underscored by the findings that *myo-3 *null mutants are nonviable as embryos whereas genetic ablation of *unc-54 *disrupts muscle structure and impairs movement but does not result in lethality [3,13,35]. Two additional transcripts, **F45G2.2 **and **Y11D7A.14**, with sequence similarity to the myosin heavy chain genes, are elevated in the M24 dataset. On the basis of strong similarity to the amino-terminal actin-binding and ATPase domain, F45G2.2 is a member of the myosin II class of striated muscle MHCs that includes *myo-3 *and *unc-54*. However, the carboxyl-terminal sequence of F45G2.2 is unusually short, with only about 100 amino acids, as opposed to the extended α-helical domain of about 1,000 amino acids in the MYO-3 and UNC-54 proteins. Because this so-called 'rod' domain drives thick filament assembly, it will be interesting to determine whether the foreshortened carboxyl-terminal region of F45G2.2 contributes to this structure. Y11D7A.14 encodes an unconventional myosin that is more distantly related to other structural myosins expressed in muscle. Potential functions for these additional myosin molecules in muscle can now be explored by genetic or RNA interference methods.

The myosin light chain proteins regulate the ATPase activity of the MHCs. Three myosin light chain genes (***mlc-1***, ***mlc-2***, and ***mlc-3***) are enriched in both datasets. Genetic data indicate that *mlc-3 *is an essential muscle component, whereas *mlc-1 *and *mlc-2 *appear to have redundant functions [3]. Paramyosin (**UNC-15**), a core component of thick filaments that interacts with MHC A (MYO-3) and MHC B (UNC-54) [32,36], shows elevated transcript levels in both M0 and M24 profiles. **UNC-45**, a highly conserved myosin binding protein and chaperone that directs assembly of these components, is enriched (Figure 9) [37].

Muscle structure and function also depend on a family of very large cytoplasmic proteins, which contain multiple fibronectin and immunoglobulin domains [38,39]. The founding member of this gene family, ***unc-22***, encodes 'twitchin', which when mutated causes constant twitching movements [3,10]. *unc-22 *is enriched in both datasets. A second member of this family, titin, adopts an elongated structure that spans half of the mammalian muscle sarcomere (from Z line to M line) and functions in myofibril assembly and elasticity [40]. *C. elegans *titin is somewhat smaller and largely localized to the thin filament or I band region of the body wall muscle sarcomere. Previous work identified a 90 kilobase gene that encodes three distinct titin isoforms in *C. elegans *[38]. We find that one of the identified titin transcripts (***ttn-1***) [39] is enriched in both M0 and M24 datasets (Table 1). Additionally, we identify ***ketn-1***, an invertebrate-specific titin-like protein [41].

Thin filaments are primarily composed of actin, troponin, and tropomyosin (Figure 9). Although actin transcripts are not enriched in the muscle datasets because of high expression in nonmuscle cells, actin genes (*act-2*, *act-3*, *act-4*, and *act-*5) are detected as expressed genes (Additional data file 2). We do see elevated expression of several actin-binding and regulatory proteins. Among these are members of the **profilin **(for example, ***pfn-3***) and **gelsolin **(for example, **K06A4.3**) families, which are proposed to regulate thin filament assembly (Table 1) [1,3]. Sarcomeres are initially assembled during embryonic development. Muscle cells add new sarcomere repeats and expand in size as the animal grows [42]. The continuous growth of the contractile apparatus during development could account for the expression of key structural components (for example, *tni-1 *and troponin) in both of the datasets. On the other hand, as noted above, genes identified in the M0 dataset may play important roles in the initial formation or organization of the sarcomere, whereas transcripts that are uniquely enriched in the M24 profile are required for sarcomere maintenance during expansion of the body wall muscle cells.

Troponin and tropomyosin form a complex that regulates actin-myosin interactions in response to calcium [1,3]. In *C. elegans*, tropomyosin is encoded by ***lev-11***, which is enriched in both datasets. Troponin is comprised of three subunits, TnI, TnT, and TnC. There are four TnI genes in the nematode, of which three (***tni-1***, ***unc-27***, and ***tni-3***) are expressed in body wall muscle [43] and each is enriched in our datasets. Similarly, all three body wall muscle expressed TnT transcripts, ***mup-2***, ***tnt-2***, and ***tnt-3***, and the TnC transcript ***pat-10 ***are enriched in both datasets (Table 1 and Figure 8).

The sarcomere is attached to the cell membrane by both dense bodies and M-lines. Many components of the dense bodies are known, including the enriched genes encoding vinculin (***deb-1***), talin (**Y71G12B.11a,b**), afadin (**W03F11.6a**) and β-1 integrin (***pat-3***). Previous studies have also identified ***unc-112 ***and ***dim-1 ***as components required for dense body assembly and maintenance, respectively [44,45]. Other enriched actin-binding proteins with potential roles in body muscle assembly include ***tag-138***, a Huntingtin-interacting protein, and ***cor-1***, which is a homolog of coronin (Table 1).

Muscle contraction is triggered by the release of intracellular calcium stores in response to neurotransmitters secreted from adjacent motor neurons at the neuromuscular junction. Cytoplasmic calcium initiates myofibril contraction and is rapidly pumped back into the sarcoplasmic reticulum via an ATP-dependent calcium channel [46]. As expected, calcium channel and calcium ion binding proteins are detected as enriched transcripts in our datasets (Table 1). For example, the ryanodine receptor, **UNC-68**, mediates the release stored calcium [47,48] and ***sca-1 ***encodes the nematode sarco-endoplasmic reticulum calcium ATPase (SERCA), which returns cytoplasmic calcium to the sarcoplasmic reticulum [46]. We also find three genes that are predicted to function as calcium sensors (**C54E10.2**, **F21A10.1**, and ***ncs-3***), as well as other components of calcium signaling including **calsequestrin **and **calmodulin **(Table 1).

### The dystrophin glycoprotein complex

In humans, Duchenne and Becker muscular dystrophies arise from mutations in a single gene encoding the large membrane-associated protein, dystrophin; these diseases are characterized by severe muscle weakening and degeneration [49]. Dystrophin is localized beneath the sarcolemma and is attached to actin filaments as well as to the dystrophin glycoprotein complex (DGC) [50]. This protein complex stabilizes the sarcolemma and prevents damage to muscle fibers induced by long-term contraction. Mutations in the *C. elegans *homolog of dystrophin (***dys-1***) lead to hyperactivity, but muscle degeneration has not been observed [51,52]. However, in *C. elegans*, as in mouse, mild MyoD (***hlh-1***) mutations in conjunction with dystrophin deficiencies act synergistically to induce muscle disassembly [50,53]; this finding suggests that *C. elegans *may be a useful model for studying these degenerative diseases. Most of the major DGC components [52], (***dys-1 ***[dystrophin], ***dyc-1 ***[CAPON], ***stn-1 ***[syntrophin], and ***sgn-1 ***[sarcoglycan]) are enriched in our *C. elegans *body wall muscle profiles; the one exception is *dyb-1 *(dystrobrevin), which is not detected. Other enriched transcripts with potentially related functions include the sarcoglycan-like genes ***sgcb-1 ***and **H22K11.4**. A GFP reporter for H22K11.4 confirms expression in body wall, anal, and vulval muscles (Figure 7b). Additionally, the M0 profile detects ***stn-2***, a second syntrophin-like gene. Given the conservation between the nematode and vertebrate homologs of the DGC components, it is probable that our datasets include transcripts for additional proteins that may be required to maintain muscle integrity.

### The neuromuscular junction

Coordinated locomotion is mediated by signaling between motor neurons and postsynaptic muscle partners. Neurotransmitters released by motor neurons activate ion channels, thereby altering the local membrane potential to regulate muscle cell activity. These transmitter-gated ionotropic receptors are clustered at the synapse and open transiently in response to neurotransmitter binding. Consistent with the importance of these molecules in facilitating synaptic signals, we find a large number of ligand-gated ion channels to be enriched in body wall muscle cells. All of the subunits of the levamisole-sensitive nicotinic acetylcholine receptor (nAChR; namely ***lev-1***, ***lev-8***, ***unc-29***, ***unc-38***, and ***unc-63***) are enriched [54-56]. Two additional uncharacterized nAChR genes, ***acr-8 ***and ***acr-16***, were also detected. We recently confirmed that *acr-16 *is an essential component of the levamisole-insensitive nAChR, thereby substantiating the utility of these data for identifying new muscle genes [16,57].

The neurotransmitter γ-aminobutyric acid (GABA) inhibits muscle activation and thus works in opposition to acetylcholine receptors. The GABA receptor gene ***unc-49 ***that mediates this response is enriched in our datasets [55]. In addition to the expected acetylcholine and GABA receptors, we also detected ionotropic receptors for other classes of neurotransmitters that have not previously been shown to regulate muscle activity directly in *C. elegans*. Prominent among these is ***mod-1***, which encodes a serotonin-gated chloride channel required for 5-hydroxytryptamine dependent inhibition of *C. elegans *locomotion [58]. Additional amine responsive ionotropic receptors include **T24D8.1 **and **Y113G7A.5**, which are activated by 5-hydroxytryptamine and tyramine, respectively, when they are expressed in *Xenopus *ooctyes (Abe N, Ringstad N, Horvitz B, personal communication). Other candidate ionotropic anion channel receptor genes include ***glc-4 ***(glutamate-gated chlorine channel) and **T27A1.4 **(similar to GABA-A receptor). Excitatory responses to glutamate could be mediated by ***glr-8 ***(glutamate-gated kainite-type ion channels).

### Transcription factors

Mammalian myogenesis is initiated by a family of HLH transcription factors that includes MyoD, myogenin, MRF-4/herculin/Myf-6, and Myf-5 [6]. In *C. elegans*, ***hlh-1 ***encodes CeMyoD and is detected in all embryogenic lineages (MS, C, D, and AB) that give rise to the body wall muscle cells [5]. Consistent with the important role of HLH-1 in early myogenesis, the *hlh-1 *transcript is enriched in both muscle datasets. Also enriched is the MADS transcription factor **UNC-120**. *unc-120 *is expressed early in embryonic development in muscle precursor cells and appears to cooperate with HLH-1 to drive muscle gene expression [59]. Additionally, we detect ***ceh-13***, a member of the HOX family of transcription factors that regulate cellular differentiation in specific body regions. *ceh-13 *expression in the embryo is limited to the anterior body wall muscles and other cell types in the head region [60].

In addition to these characterized transcription factors, we detect eight putative nuclear hormone receptors (NHRs; Table 1). In *C. elegans*, the NHR family consists of about 280 receptors that are presumptively regulated by lipophilic hormones to control a variety of processes from sex determination to lifespan [61]. Functions for the NHR transcripts showing enriched expression in our muscle datasets have not been characterized. However, recent studies of an NHR gene, *nhr-40*, which is detected as an expressed gene in both datasets, have identified a key role in muscle development; mutations in *nhr-40 *result in late embryonic/early larval arrest with irregular development of body wall muscle cells and uncoordinated locomotion [62]. The significance of this class of transcription factors to vertebrate muscle development is uncertain because the majority of nematode NHR sequences are not well conserved. On the other hand, the absence of clear mammalian homologs offers the possibility of developing phylum-specific nematocides that target these diverged NHR proteins.

A total of 38 transcription factors are enriched in muscle cells in these experiments (Table 1). The functions of a majority of these transcription factors in muscle development have not been explored. It is also interesting that most of these transcription factors are selectively enriched in either the M0 (13) or M24 (18) dataset (Table 1). This finding could mean that muscle development is orchestrated by a diverse array of transcription factors with functions that are specifically required in either early muscle precursor cells or later to regulate expression of terminal muscle differentiation.

## Discussion

Our application of MAPCeL to *C. elegans *embryonic body wall muscle has generated a high-quality gene expression profile that defines the embryonic muscle transcriptome. We base this conclusion on five observations. First, we have successfully isolated *myo-3*::GFP labeled muscle cells directly from embryos and determined that transcriptional profiles obtained from these cells are reproducible. Second, sorting of cultured muscle cell populations yields similarly reproducible data and a transcriptional profile consistent with more fully differentiated muscle cells. Third, our muscle-enriched gene lists are largely distinct from profiles of other nonmuscle cell types from *C. elegans *(Additional data file 6). Conversely (fourth), the MAPCeL datasets show substantial overlap with an independent experiment in which most embryonic blastomeres were converted to body wall muscle-like cell fates *in vivo *and profiled on the Affymetrix array (Additional data file 10) [7]. Fifth, transgenic GFP reporters generated in this study confirmed that a majority of muscle-enriched genes were expressed in muscle *in vivo*. Based on these observations, we suggest that our results provide a comprehensive profile of gene expression in developing *C. elegans *body wall muscle cells. Moreover, the common group of 592 genes in these microarray profiles that are also specifically upregulated with the induction of embryonic muscle differentation are likely to comprise a core group of genes with fundamental roles in myogenesis. An additional 719 genes are identified that may also contribute substantially to the myogenic program (Figure 6). These lists can now be exploited in future work for studies of muscle development, myofibril assembly, and muscle function (Additional data file 8).

The strong concurrence of our data with known or predicted muscle proteins (Table 1) underscores the potential utility of these MAPCeL profiles for identifying candidate muscle genes that can now be tested by genetic methods in this model organism. Examples include F45G2.2, an atypical member of the myosin II family that shows strong homology to the head region of known *C. elegans *body wall muscle myosin heavy chain genes (for instance, *myo-3*) but for which a muscle function has not previously been described. Muscle enrichment of *tag-138 *is intriguing because its vertebrate homolog HIP1 (Huntingtin interacting protein 1) mediates receptor endocytosis, is highly expressed in a variety of human tumors, and may function as an oncogene [63]. Mutations affecting components of the DGC result in human muscular dystrophies, and similar genetic defects in *C. elegans *also disrupt body wall muscle structure and function [49-51]. Our muscle datasets have detected potential additional members of the DGC, namely the sarcoglycan-like genes *sgcb-1 *and H22K11.4 and a syntrophin-like gene, *stn-2*, which can now be experimentally tested for related functions. In addition to confirming muscle enrichment of known neurotransmitter-gated ion channels, these data have also identified several new possible receptors for modulating muscle activity. Indeed, enrichment of the nAChR subunit gene *acr-16 *in the M24 dataset led to physiological experiments that confirmed a key role for the ACR-16 receptor in acetylcholine-evoked muscle excitation [16,57]. These positive results, which are based on a limited survey of the muscle genes in our datasets, suggest that a more detailed analysis of these gene lists should reveal a substantial number of additional muscle functional genes.

The evolutionary conservation of sarcomere structure and function from *C. elegans *to mammals has made the nematode an attractive model for studies of muscle development [1-3]. Past work in the worm has been useful for understanding general concepts of myosin filament assembly [8,32,37], the molecular mechanisms that underlie the transduction of sarcomere contractile force to connected tissues [2,39] and the evolution of striated muscle specification [7]. Our gene expression profiles should similarly shed light on myogenesis in other species, including humans. Approximately 60% of transcripts enriched in at least one of the embryonic body wall muscle datasets (787/1,312) are conserved in the human genome (BLAST = e-10; Additional data file 13). Although many of the transcripts in this list encode proteins with well established roles in mammalian muscle (for example, myosin heavy chain), potential muscle functions for a substantial number of additional proteins have not been defined. Of particular interest are 32 transcripts encoding proteins with no known function in any organism ('uncharacterized conserved protein'; Additional data file 13). Our data strongly suggest that many of these novel proteins play important roles in myogenesis or muscle function and serve as ripe targets for future studies, both in *C. elegans *and in other animals.

We view this study as a starting point for determining how the *C. elegans *muscle cell transcriptome regulates myogenesis. Our approach provides a temporal component by profiling both nascent and differentiated muscle cells, and we have examples from our data in which transcript appearance mimics the known order of protein assembly within the sarcomere (for example, UNC-54). It may be possible to enhance the temporal resolution of MAPCeL by profiling embryos expressing a series of muscle reporters that come on at successive developmental time points. These data could potentially provide clues as to how the transcriptome temporally orchestrates myogenesis to assemble myofibrils into functional sarcomeres. Profiling data collected from subsets of embryonic muscle (anal and pharyngeal muscles) could also reveal gene sets with specialized functions in these different muscle types.

The embryonic muscle profiles described in this paper show substantial overlap (about 600 genes) with an independent microarray dataset obtained from embryonic muscle cells induced by ectopic expression of the MRF related transcription factor HLH-1 (Figure 6 and Additional data file 10) [7]. Our MAPCeL datasets show less similarity, however (about 250 common genes), with a profile of larval muscles obtained using the mRNA tagging strategy (Additional data file 8). In this approach, an epitope-tagged poly-A binding protein was used to specifically pull down body wall muscle transcripts from L1 stage larvae [25]. It seems unlikely that this result can be fully attributed to differences in developmental age (embryonic versus larval) because reporter gene constructs for genes in our MAPCeL datasets showed post-embryonic expression in one or more muscle types (Figure 7). One potential explanation for this disparity is the relative sensitivity of the two profiling approaches used to generate these data.

A recent modification of the mRNA tagging strategy that reduces background RNA could yield a deeper dataset of larval muscle enriched transcripts [24]. The use of the mRNA tagging method to profile larval muscles is necessary because post-embryonic cells are not readily accessible to MAPCeL analysis [14,17]. mRNA tagging affords the additional benefit of providing sharply defined temporal profiles of gene expression that could potentially identify transcriptional cascades of genes that control muscle differentiation and growth during this period. Finally, mRNA tagging profiles of aging body wall muscle cells could reveal transcriptionally regulated genes associated with sarcopenia, an evolutionarily conserved process in which body wall muscles in *C. elegans *exhibit morphological disorganization and functional decline that resembles the progressive age-related muscle atrophy that occurs in mammals [64]. In this context, it will be interesting to compare these gene expression data with MAPCeL profiles of embryonic muscle cells maintained in culture for prolonged periods to potentially distinguish between autonomous versus environmentally induced aging processes.

## Conclusion

We used MAPCeL to generate comprehensive descriptions of gene expression in developing *C. elegans *embryonic body wall muscle cells. In addition to detecting known muscle genes, our MAPCeL dataset has also identified a large number of previously uncharacterized transcripts with potentially important roles in muscle development or structure. Therefore, this work defines a basic muscle transcriptome that can lead to new discoveries into how these genes are deployed to drive myofibril assembly and function.

## Materials and methods

### Nematode strains

Nematode strains were maintained at 20°C to 25°C using standard culture methods [9]. Strains used for microarray experiments were the N2 wild-type isolate and PD4251 (*ccIs4251 *and *myo-3*::GFP) [65]. Transgenic GFP reporter lines generated from muscle-enriched genes are listed in Additional data file 11.

### Generating transgenic promoter::GFP strains to validate microarray data

Promoter regions for GFP reporter constructs were either cloned into the BamHI site of pPD95.67 (Fire Lab Vector Kit 1995, Addgene Inc., Cambridge, MA, USA) or fused to GFP in this same vector using the sequence overlap extension (SOE) method [66]. Transgenic animals were created by microinjection of a mixture including 23 μl (5 to 10 ng/μl) of SOE reaction with 3 μl (60 ng/μl) pRF4 [*rol-6(d)*] as a co-injectable marker. There were two exceptions to this method (H22K11.4::GFP and E02H4.3::GFP), which were generated using biolistic transformation. For these two reporters, promoter regions were cloned into HindIII (H22K11.4)/PstI (E02H4.3) and XmaI sites of pPD95.75 GFP plasmid (1995 Fire Vector Kit) along with the *unc-119 *minigene. Microparticle bombardment was conducted as previously described [14]. At least two stable extrachromosomal transgenic lines were generated for each construct and examined multiple times at all developmental stages for muscle expression. Lines that gave reproducible expression in at least one *myo-3*::GFP expressed muscle cell type (body wall, defecation, and vulval) were scored as positive; the exact GFP pattern description for each transgenic strain is indicated in Additional data file 11.

### Cell culture

*C. elegans *embryos were dissociated for FACS isolation of freshly dissociated *myo-3*::GFP muscle cells (M0; see below). Embryonic cells were also cultured for 24 hours, as described previously [14], to generate the M24 *myo-3*::GFP muscle cells for FACS isolation [17].

### FACS analysis

FACS experiments were conducted on a FACStar Plus flow cytometer (Becton Dickinson, San Jose, CA, USA) equipped with a 488 nm argon laser. Emission filters were 530 ± 30 nm for GFP and 585 ± 22 nm for propidium iodide (PI). The machine was flushed with egg buffer [17] and light scattering parameters calibrated with 2 μm beads. The rate of sorting was 4,000 to 5,000 cells per second through a 70 μm nozzle.

*myo-3*::GFP labeled cells were isolated from freshly dissociated embryos as follows. Chitinase-treated embryos were dissociated by gentle re-suspension in egg buffer and passed through a 5 μm durapore filter to remove intact embryos and debris. Cells were counted on a hemocytometer and diluted to a concentration of about 10 million cells/ml. PI was added to a final concentration of 5 μg/ml. FACS parameters for setting the GFP positive gate were established as follows. First, wild-type non-GFP embryonic cells (from N2) were used to identify a population of autofluorescent cells. These gut cells fluoresce in both the red (PI) and green (GFP) channels and can be visualized along the diagonal axis of the scatter plot in Figure 2c. Next, gating criteria for non-viable cells were determined by sorting wildtype cells stained with PI. These scatter plots were compared with a profile of *myo-3*::GFP embryonic cells to define the GFP sorting window (Figure 2d). Viable GFP positive muscle cells were then gated according to light scattering parameters (Size, x-axis; and granularity, y-axis; Figure 2e) to isolate a subpopulation of cells that excludes large clumps and small debris. Direct visual inspection of *myo-3*::GFP cells isolated according to these criteria confirmed that GFP positive cells comprise about 90% of the total population for an overall sixfold enrichment (90%/15%) of *myo-3*::GFP cells after FACS in comparison to the intact embryo (Figure 2f). A typical sort yielded about 300,000 *myo-3*::GFP cells.

Cultured *myo-3*::GFP muscle cells were isolated as previously described [14,16]. Reference datasets were generated from all viable embryonic cells sorted either immediately after dissociation (R0) or after 24 hours in culture (R24).

A detailed bench protocol can be found in the online WormBook, protocol 32 [67].

### RNA isolation, amplification, and hybridization

RNA was extracted from FACS isolated GFP positive muscle cells for comparison with reference RNA obtained from a sorted population containing all viable embryonic cells. RNA was isolated using a Micro-RNA isolation kit (Strategene, La Jolla, CA, USA), and 100 ng of total RNA was amplified with a modification of the Affymetrix GeneChip Eukaryotic Small Sample Labeling Protocol (Affymetrix Inc., Santa Clara, CA, USA), as previously described [16]. aRNA was biotin-labeled during the second round of amplification using the BioArray High Yield RNA transcript Labeling Kit (Enzo, New York, NY, USA). Fifteen micrograms of biotinylated aRNA was fragmented for hybridization to the Affymetrix *C. elegans *array. RNA quality was assessed after fragmentation with the Agilent Bioanalyzer (Agilent, Santa Clara, CA, USA).

### Data analysis

All experiments were performed in triplicate with the exception of the cultured cell reference dataset (R24), which was generated from four independent experiments [14]. Raw signal intensities were scaled for interchip comparisons using Affymetrix MAS 5.0. Transcripts were deemed 'present' if assigned a 'present' call by Affymetrix MAS 5.0 in a majority of replicates for a given sample (two-thirds for M0 muscle, two-thirds for M24 muscle, two-thirds for reference R0, and three-quarters for reference R24). Intensity values were normalized using robust multiarray analysis (Additional data file 1), available through GeneTraffic (Iobion), and statistical analysis was performed with significance analysis of microarray software [68]. A two-class unpaired analysis was performed to identify genes that are elevated 1.7-fold or greater when compared with the reference for each dataset, at a false discovery rate of 1.8% or less for M0 and 1.2% or less for the M24 datasets. For the M0 muscle dataset, 770 genes were considered significantly enriched whereas 937 genes were enriched in the M24 muscle dataset (Additional data file 4).

For the M0 and M24 datasets, the list of present calls as defined above was modified to exclude transcripts that can be attributed to the small fraction (about 10%) of non-GFP cells in the sorted preparations. Transcripts from these contaminating cells were identified as genes showing relative enrichment in the reference datasets obtained from all embryonic cells (978 for M0 muscle and 961 for M24 muscle). A limited number of transcripts (54 in M0 muscle and 190 in M24 muscle) initially flagged as 'absent' by MAS 5.0 on the basis of perfect match versus mismatch signals were restored to the expressed gene files because significance analysis of microarray analysis scores these genes as enriched in the muscle samples relative to reference. These considerations identified 5,170 expressed genes in the M0 muscle dataset and 6,088 expressed genes in the M24 muscle dataset (Additional data file 2).

The data discussed in this publication have been deposited in the National Center for Biotechnology Information's Gene Expression Omnibus [69,70] and are accessible through Gene Expression Omnibus series accession numbers GSE8462 (M0 and M24) and GSE8231 (HLH-1).

### Bioinformatic search for known muscle expressed transcripts

We utilized Perl scripts and hand annotation to identify all transcripts annotated in WormBase release WS170 [71] as expressed in body wall or defecation muscle cells. The cell identity column was searched using the following keywords: body wall muscle, BWM, anal sphincter, anal depressor, head muscle, anal muscle, all cells, and ubiquitous. This analysis identified a total of 925 muscle-expressed genes. A survey of recent literature using Textpresso (keywords: body, muscle, and expression) revealed an additional 78 genes that met these criteria for a total of 1,003 muscle-expressed genes.

### Global analysis of microarray data

Annotation scripts were used to extract information from WormBase using the Affy ID and cosmid name as previously described [14].

## Abbreviations

DGC, dystrophin glycoprotein complex; FACS, fluorescence-activated cell sorting; GABA, γ-aminobutyric acid; GFP, green fluorescent protein; HLH, helix-loop-helix; MAPCeL, microarray profiling of *C. elegans *cells; MHC, myosin heavy chain; MRF, myogenic regulatory factor; nAChR, nicotinic acetylcholine receptor; NHR, nuclear hormone receptor; PI, propidium iodide; SOE, sequence overlap extension; UNC, uncoordinated.

## Authors' contributions

RMF collected the M0, M24, and R0 microarray profiles; performed validation experiments; generated and scored GFP reporters for H22K11.4 and E02H4.3; and drafted the manuscript. JDW compiled expression data from WormBase and aided in data analysis. SEV helped generate GFP reporters and aided in data analysis. JM designed and built GFP reporter genes using the SOE method. TMB generated transgenic GFP lines and TF analyzed the HLH-1 induced microarray data generated in the Krause laboratory. MK directed experiments in the Krause lab, designed GFP reporters, scored GFP expression, and helped to write the manuscript. DMM oversaw experiments conducted in the Miller laboratory and helped to draft the manuscript.

## Additional data files

The following additional data are available with the online version of this paper. Additional data file 1 provides a complete list of RMA normalized intensity values from 0 hours (M0) and 24 hours (M24) datasets. Additional data file 2 provides all expressed gene lists. Additional data file 3 provides comparisons of expressed gene lists (total reference expressed genes [R0 + R24] versus total muscle expressed genes [M0 + M24]; M0 versus M24; total unique genes). Additional data file 4 summarizes M0 and M24 enriched genes, plus the total unique genes from both lists (total muscle), and provides comparisons of these lists. Additional data file 5 lists genes depleted in M0 and M24 and provides a comparison of those lists. Additional data file 6 provides a master annotation file of all probesets represented on the *C. elegans *Affymetrix microarray. Additional data file 7 provides a master annotation file of all genes represented on the *C. elegans *Affymetrix microarray. Additional data file 8 provides comparisons with other datasets (Germline enriched or mRNA tagging isolated intestine enriched [GI] versus total muscle, L1 mRNA tagging isolated muscle transcripts versus total muscle). Additional data file 9 provides a comparison of total muscle enriched genes versus embryonic A-class (EA) and total muscle enriched genes versus embryonic pan-neural (EP). Additional data file 10 provides a comparison of total muscle dataset with HLH-1 induced transcripts. Additional data file 11 provides a full list of GFP reporters generated. Additional data file 12 provides a table of muscle structural genes. Additional data file 13 summarizes total muscle genes with human homologs.

## Supplementary Material

Additional data file 1 Provided is the complete list of RMA normalized intensity values from 0 hour (M0) and 24 hour (M24) datasets. Click here for file

Additional data file 2 All expressed gene lists are provided. Click here for file

Additional data file 3 Provided are comparisons of expressed gene lists (total reference expressed genes [R0 + R24] versus total muscle expressed genes [M0 + M24]; M0 versus M24; total unique genes). Click here for file

Additional data file 4 Summarized are M0 and M24 enriched genes, plus the total unique genes from both lists (total muscle), and comparisons of these lists are provided. Click here for file

Additional data file 5 Genes depleted in M0 and M24 are summarized, and a comparison of those lists is provided. Click here for file

Additional data file 6 Provided is a master annotation file of all probe sets represented on the *C. elegans *Affymetrix microarray, based on WormBase Release WS170. Annotation of known muscle expressed genes is included. Click here for file

Additional data file 7 Provided is a master annotation file of all genes represented on the *C. elegans *Affymetrix microarray, based on WormBase Release WS170. Annotation of known muscle expressed genes is included. Click here for file

Additional data file 8 Provided are comparisons with other datasets (germline enriched or mRNA tagging isolated intestine enriched [GI] versus total muscle, L1 mRNA-tagging isolated muscle transcripts versus total muscle). Reannotated mRNA-tagging data for genes represented on the *C. elegans *Affymetrix microarray are provided. Also included are embryonic pan-neural (EP), embryonic A-class (EA), and GI datasets. Click here for file

Additional data file 9 Provided is a comparison of total muscle enriched genes versus embryonic A-class (EA) and total muscle enriched genes versus embryonic pan-neural (EP) Click here for file

Additional data file 10 Presented is a comparison of the total muscle dataset with HLH-1 induced transcripts. Click here for file

Additional data file 11 Provided is the full list of GFP reporters generated. Summarized expression patterns and primer sets used to generate each promoter::GFP are included. Click here for file

Additional data file 12 Presented is a table of muscle structural genes. Click here for file

Additional data file 13 Summarized are the total muscle genes with human homologs. Click here for file

## References

[B1] Waterston RH, Wood WB (1988). Muscle.. The Nematode Caenorhabditis elegans.

[B2] Moerman DG, Williams BD Sarcomere assembly in *C. elegans *muscle.. WormBook.

[B3] Moerman DG, Fire A, Riddle D, Blumenthal T, Meyer B, Priess J (1997). Muscle: structure, function and development.. C elegans II.

[B4] Berkes CA, Tapscott SJ (2005). MyoD and the transcriptional control of myogenesis.. Semin Cell Dev Biol.

[B5] Krause M, Fire A, Harrison SW, Priess J, Weintraub H (1990). CeMyoD accumulation defines the body wall muscle cell fate during C. elegans embryogenesis.. Cell.

[B6] Fukushige T, Krause M (2005). The myogenic potency of HLH-1 reveals wide-spread developmental plasticity in early C. elegans embryos.. Development.

[B7] Fukushige T, Brodigan TM, Schriefer LA, Waterston RH, Krause M (2006). Defining the transcriptional redundancy of early bodywall muscle development in *C. elegans*: evidence for a unified theory of animal muscle development.. Genes Dev.

[B8] Miller DM, Ortiz I, Berliner GC, Epstein HF (1983). Differential localization of two myosins within nematode thick filaments.. Cell.

[B9] Brenner S (1974). The genetics of *Caenorhabditis elegans*.. Genetics.

[B10] Waterston RH, Thomson JN, Brenner S (1980). Mutants with altered muscle structure of *Caenorhabditis elegans*.. Dev Biol.

[B11] Williams BD, Waterston RH (1994). Genes critical for muscle development and function in *Caenorhabditis elegans *identified through lethal mutations.. J Cell Biol.

[B12] Zengel JM, Epstein HF (1980). Identification of genetic elements associated with muscle structure in the nematode *Caenorhabditis elegans*.. Cell Motil.

[B13] Waterston RH (1989). The minor myosin heavy chain, mhcA, of *Caenorhabditis elegans *is necessary for the initiation of thick filament assembly.. EMBO J.

[B14] Fox RM, Von Stetina SE, Barlow SJ, Shaffer C, Olszewski KL, Moore JH, Dupuy D, Vidal M, Miller DM (2005). A gene expression fingerprint of *C. elegans *embryonic motor neurons.. BMC Genomics.

[B15] Fire A, Waterston RH (1989). Proper expression of myosin genes in transgenic nematodes.. EMBO J.

[B16] Touroutine D, Fox RM, Von Stetina SE, Burdina A, Miller DM, Richmond JE (2005). acr-16 encodes an essential subunit of the levamisole-resistant nicotinic receptor at the *Caenorhabditis elegans *neuromuscular junction.. J Biol Chem.

[B17] Christensen M, Estevez A, Yin X, Fox R, Morrison R, McDonnell M, Gleason C, Miller DM, Strange K (2002). A primary culture system for functional analysis of C. elegans neurons and muscle cells.. Neuron.

[B18] Ardizzi JP, Epstein HF (1987). Immunochemical localization of myosin heavy chain isoforms and paramyosin in developmentally and structurally diverse muscle cell types of the nematode *Caenorhabditis elegans*.. J Cell Biol.

[B19] Waterston RH, Epstein HF, Brenner S (1974). Paramyosin of *Caenorhabditis elegans*.. J Mol Biol.

[B20] Miller DM, Stockdale F, Karn J (1986). Immunological identification of the genes encoding the four myosin heavy chains of *Caenorhabditis elegans*.. Proc Natl Acad Sci USA.

[B21] Nonet ML, Saifee O, Zhao H, Rand JB, Wei L (1998). Synaptic transmission deficits in *Caenorhabditis elegans *synaptobrevin mutants.. J Neurosci.

[B22] Worm Base. http://www.wormbase.org.

[B23] Pauli F, Liu Y, Kim YA, Chen PJ, Kim SK (2006). Chromosomal clustering and GATA transcriptional regulation of intestine-expressed genes in *C. elegans*.. Development.

[B24] Von Stetina SE, Watson JD, Fox RM, Olszewski KL, Spencer WC, Roy PJ, Miller DM (2007). Cell-specific microarray profiling experiments reveal a comprehensive picture of gene expression in the *C. elegans *nervous system.. Genome Biol.

[B25] Roy PJ, Stuart JM, Lund J, Kim SK (2002). Chromosomal clustering of muscle-expressed genes in *Caenorhabditis elegans*.. Nature.

[B26] Gettner SN, Kenyon C, Reichardt LF (1995). Characterization of beta pat-3 heterodimers, a family of essential integrin receptors in *C. elegans*.. J Cell Biol.

[B27] Lin X, Qadota H, Moerman DG, Williams BD (2003). C. elegans PAT-6/actopaxin plays a critical role in the assembly of integrin adhesion complexes in vivo.. Curr Biol.

[B28] Hammarlund M, Davis WS, Jorgensen EM (2000). Mutations in beta-spectrin disrupt axon outgrowth and sarcomere structure.. J Cell Biol.

[B29] Szewczyk NJ, Hartman JJ, Barmada SJ, Jacobson LA (2000). Genetic defects in acetylcholine signalling promote protein degradation in muscle cells of *Caenorhabditis elegans*.. J Cell Sci.

[B30] Francis GR, Waterston RH (1985). Muscle organization in *Caenorhabditis elegans*: localization of proteins implicated in thin filament attachment and I-band organization.. J Cell Biol.

[B31] Bartnik E, Osborn M, Weber K (1986). Intermediate filaments in muscle and epithelial cells of nematodes.. J Cell Biol.

[B32] Epstein HF, Casey DL, Ortiz I (1993). Myosin and paramyosin of *Caenorhabditis elegans *embryos assemble into nascent structures distinct from thick filaments and multi-filament assemblages.. J Cell Biol.

[B33] Epstein HF, Ortiz I, Mackinnon LA (1986). The alteration of myosin isoform compartmentation in specific mutants of *Caenorhabditis elegans*.. J Cell Biol.

[B34] Epstein HF (1985). Myosins A & B in the organization of myofilaments.. Adv Exp Med Biol.

[B35] Moerman DG, Plurad S, Waterston RH (1982). Mutations in the unc-54 myosin heavy chain gene of *Caenorhabditis elegans *that alter contractility but not muscle structure.. Cell.

[B36] Hoppe PE, Waterston RH (1996). Hydrophobicity variations along the surface of the coiled-coil rod may mediate striated muscle myosin assembly in *Caenorhabditis elegans*.. J Cell Biol.

[B37] Hutagalung AH, Landsverk ML, Price MG, Epstein HF (2002). The UCS family of myosin chaperones.. J Cell Sci.

[B38] Flaherty DB, Gernert KM, Shmeleva N, Tang X, Mercer KB, Borodovsky M, Benian GM (2002). Titins in *C. elegans *with unusual features: coiled-coil domains, novel regulation of kinase activity and two new possible elastic regions.. J Mol Biol.

[B39] Ferrara TM, Flaherty DB, Benian GM (2005). Titin/connectin-related proteins in *C. elegans*: a review and new findings.. J Muscle Res Cell Motil.

[B40] Gregorio CC, Granzier H, Sorimachi H, Labeit S (1999). Muscle assembly: a titanic achievement?. Curr Opin Cell Biol.

[B41] Ono K, Yu R, Mohri K, Ono S (2006). *Caenorhabditis elegans *kettin, a large immunoglobulin-like repeat protein, binds to filamentous actin and provides mechanical stability to the contractile apparatuses in body wall muscle.. Mol Biol Cell.

[B42] Mackenzie JM, Garcea RL, Zengel JM, Epstein HF (1978). Muscle development in *Caenorhabditis elegans*: mutants exhibiting retarded sarcomere construction.. Cell.

[B43] Burkeen AK, Maday SL, Rybicka KK, Sulcove JA, Ward J, Huang MM, Barstead R, Franzini-Armstrong C, Allen TS (2004). Disruption of *Caenorhabditis elegans *muscle structure and function caused by mutation of troponin I.. Biophys J.

[B44] Rogalski TM, Mullen GP, Gilbert MM, Williams BD, Moerman DG (2000). The UNC-112 gene in *Caenorhabditis elegans *encodes a novel component of cell-matrix adhesion structures required for integrin localization in the muscle cell membrane.. J Cell Biol.

[B45] Rogalski TM, Gilbert MM, Devenport D, Norman KR, Moerman DG (2003). DIM-1, a novel immunoglobulin superfamily protein in *Caenorhabditis elegans*, is necessary for maintaining bodywall muscle integrity.. Genetics.

[B46] Zwaal RR, Van Baelen K, Groenen JT, van Geel A, Rottiers V, Kaletta T, Dode L, Raeymaekers L, Wuytack F, Bogaert T (2001). The sarco-endoplasmic reticulum Ca^2+ ^ATPase is required for development and muscle function in *Caenorhabditis elegans*.. J Biol Chem.

[B47] Maryon EB, Saari B, Anderson P (1998). Muscle-specific functions of ryanodine receptor channels in *Caenorhabditis elegans*.. J Cell Sci.

[B48] Maryon EB, Coronado R, Anderson P (1996). unc-68 encodes a ryanodine receptor involved in regulating *C. elegans *body-wall muscle contraction.. J Cell Biol.

[B49] Muntoni F, Torelli S, Ferlini A (2003). Dystrophin and mutations: one gene, several proteins, multiple phenotypes.. Lancet Neurol.

[B50] Gieseler K, Grisoni K, Segalat L (2000). Genetic suppression of phenotypes arising from mutations in dystrophin-related genes in *Caenorhabditis elegans*.. Curr Biol.

[B51] Segalat L (2002). Dystrophin and functionally related proteins in the nematode *Caenorhabditis elegans*.. Neuromuscul Disord.

[B52] Grisoni K, Martin E, Gieseler K, Mariol MC, Segalat L (2002). Genetic evidence for a dystrophin-glycoprotein complex (DGC) in *Caenorhabditis elegans*.. Gene.

[B53] Megeney LA, Kablar B, Garrett K, Anderson JE, Rudnicki MA (1996). MyoD is required for myogenic stem cell function in adult skeletal muscle.. Genes Dev.

[B54] Culetto E, Baylis HA, Richmond JE, Jones AK, Fleming JT, Squire MD, Lewis JA, Sattelle DB (2004). The *Caenorhabditis elegans *unc-63 gene encodes a levamisole-sensitive nicotinic acetylcholine receptor alpha subunit.. J Biol Chem.

[B55] Richmond JE, Jorgensen EM (1999). One GABA and two acetylcholine receptors function at the *C. elegans *neuromuscular junction.. Nat Neurosci.

[B56] Towers PR, Edwards B, Richmond JE, Sattelle DB (2005). The *Caenorhabditis elegans *lev-8 gene encodes a novel type of nicotinic acetylcholine receptor alpha subunit.. J Neurochem.

[B57] Francis MM, Evans SP, Jensen M, Madsen DM, Mancuso J, Norman KR, Maricq AV (2005). The Ror receptor tyrosine kinase CAM-1 is required for ACR-16-mediated synaptic transmission at the *C. elegans *neuromuscular junction.. Neuron.

[B58] Ranganathan R, Cannon SC, Horvitz HR (2000). MOD-1 is a serotonin-gated chloride channel that modulates locomotory behaviour in *C. elegans*.. Nature.

[B59] Dichoso D, Brodigan T, Chwoe KY, Lee JS, Llacer R, Park M, Corsi AK, Kostas SA, Fire A, Ahnn J (2000). The MADS-Box factor CeMEF2 is not essential for *Caenorhabditis elegans *myogenesis and development.. Dev Biol.

[B60] Brunschwig K, Wittmann C, Schnabel R, Burglin TR, Tobler H, Muller F (1999). Anterior organization of the *Caenorhabditis elegans *embryo by the labial-like Hox gene ceh-13.. Development.

[B61] Gissendanner CR, Crossgrove K, Kraus KA, Maina CV, Sluder AE (2004). Expression and function of conserved nuclear receptor genes in *Caenorhabditis elegans*.. Dev Biol.

[B62] Brozova E, Simeckova K, Kostrouch Z, Rall JE, Kostrouchova M (2006). NHR-40, a *Caenorhabditis elegans *supplementary nuclear receptor, regulates embryonic and early larval development.. Mech Dev.

[B63] Hyun TS, Ross TS (2004). HIP1: trafficking roles and regulation of tumorigenesis.. Trends Mol Med.

[B64] Herndon LA, Schmeissner PJ, Dudaronek JM, Brown PA, Listner KM, Sakano Y, Paupard MC, Hall DH, Driscoll M (2002). Stochastic and genetic factors influence tissue-specific decline in ageing *C. elegans*.. Nature.

[B65] Fire A, Ahnn J, Kelly W, Harfe B, Kostas S, Hsieh J, Hsu M, Xu S, Chalfie M, Kain S (1998). GFP applications in *C. elegans*.. GFP Strategies and Applications.

[B66] Hobert O (2002). PCR fusion-based approach to create reporter gene constructs for expression analysis in transgenic *C. elegans*.. Biotechniques.

[B67] Shaham S, editor Methods in cell biology.. WormBook.

[B68] SAM: Significant Analysis of Micoarrays. http://www-stat.stanford.edu/~tibs/SAM/.

[B69] Barrett T, Edgar R (2006). Gene expression omnibus: microarray data storage, submission, retrieval, and analysis.. Methods Enzymol.

[B70] Edgar R, Domrachev M, Lash AE (2002). Gene Expression Omnibus: NCBI gene expression and hybridization array data repository.. Nucleic Acids Res.

[B71] WormBase Release WS170. http://ws170.wormbase.org.

